# Supplementary material for: Complexome profiling of the Chlamydomonas psb28 mutant reveals TEF5 as an early PSII assembly factor
Source: Plant Cell. 2025 Mar 17;37(6):koaf055. doi: 10.1093/plcell/koaf055 (PMC12164590; doi:10.1093/plcell/koaf055)
Supplement: koaf055_Supplementary_Data [file koaf055_supplementary_data.zip › psb28_interactive.zip!/plots/intensity/qualityPlots/peptides/LPA2_Cre02.g105650.t1.2.html]

### Cre02.g105650.t1.2

Trivial name: LPA2  
SeqCoverage [median]: 35.40  
Mass: 18.78 kDa  
  
 

| Sequence | MissedCleavage | Mass [Da] | wtIntensity | muIntensity | Score |
| --- | --- | --- | --- | --- | --- |
| EAEAEASTSKPSGDGDAAALEALEAR | 1 | 2545.18 | 4300.05 | 6622.08 | 121.38 |
| GFGSETAK | 0 | 795.38 | 363.65 | 0.00 | 58.98 |
| QKEAEAEASTSKPSGDGDAAALEALEAR | 2 | 2801.33 | 1615.89 | 1037.24 | 59.28 |
| SKGFGSETAK | 1 | 1010.50 | 1620.58 | 0.00 | 112.36 |
| TGKLPGQIQK | 1 | 1068.63 | 1885.09 | 1331.99 | 68.89 |
| VKVNAPAVDVATGK | 1 | 1367.78 | 17228.56 | 17162.26 | 171.36 |
| VNAPAVDVATGK | 0 | 1140.61 | 8892.65 | 8909.87 | 106.40 |
|  |  |  | 35906.5 | 35063.4 |  |
